# Polymorphisms in the *F8* Gene and MHC-II Variants as Risk Factors for the Development of Inhibitory Anti-Factor VIII Antibodies during the Treatment of Hemophilia A: A Computational Assessment

**DOI:** 10.1371/journal.pcbi.1003066

**Published:** 2013-05-16

**Authors:** Gouri Shankar Pandey, Chen Yanover, Tom E. Howard, Zuben E. Sauna

**Affiliations:** 1 Laboratory of Hemostasis, Division of Hematology, Center for Biologics Evaluation and Research, Food and Drug Administration, Bethesda, Maryland, United States of America; 2 Program in Computational Biology, Fred Hutchinson Cancer Research Center, Seattle, Washington, United States of America; 3 Department of Pathology and Laboratory Medicine, Veterans Affairs Greater Los Angeles Healthcare System, Los Angeles, California, United States of America; Technical University of Denmark, Denmark

## Abstract

The development of neutralizing anti-drug-antibodies to the Factor VIII protein-therapeutic is currently the most significant impediment to the effective management of hemophilia A. Common non-synonymous single nucleotide polymorphisms (ns-SNPs) in the *F8* gene occur as six haplotypes in the human population (denoted H1 to H6) of which H3 and H4 have been associated with an increased risk of developing anti-drug antibodies. There is evidence that CD4+ T-cell response is essential for the development of anti-drug antibodies and such a response requires the presentation of the peptides by the MHC-class-II (MHC-II) molecules of the patient. We measured the binding and half-life of peptide-MHC-II complexes using synthetic peptides from regions of the Factor VIII protein where ns-SNPs occur and showed that these wild type peptides form stable complexes with six common MHC-II alleles, representing 46.5% of the North American population. Next, we compared the affinities computed by NetMHCIIpan, a neural network-based algorithm for MHC-II peptide binding prediction, to the experimentally measured values and concluded that these are in good agreement (area under the ROC-curve of 0.778 to 0.972 for the six MHC-II variants). Using a computational binding predictor, we were able to expand our analysis to (a) include all wild type peptides spanning each polymorphic position; and (b) consider more MHC-II variants, thus allowing for a better estimation of the risk for clinical manifestation of anti-drug antibodies in the entire population (or a specific sub-population). Analysis of these computational data confirmed that peptides which have the wild type sequence at positions where the polymorphisms associated with haplotypes H3, H4 and H5 occur bind MHC-II proteins significantly more than a negative control. Taken together, the experimental and computational results suggest that wild type peptides from polymorphic regions of FVIII constitute potential T-cell epitopes and thus could explain the increased incidence of anti-drug antibodies in hemophilia A patients with haplotypes H3 and H4.

## Author Summary

The development of anti-drug antibodies to therapeutic proteins is a significant impediment to development and licensure of therapeutic proteins and limits their clinical utility. The development of such antibodies requires CD4+ T-cell activation, which is mediated by the recognition of epitopes presented by MHC class-II (MHC-II) molecules. Here, we use experimental measurements and computational predictions of peptide-MHC-II affinities to study the clinical observation that African-American hemophilia A patients have a higher incidence of anti-drug antibodies to Factor VIII than Caucasian patients. Specifically, we used the experimental data to select and validate a computational prediction method which, in turn, allowed us to expand our analysis to a larger repertoire of peptide-MHC-II complexes. We showed that wild type peptides spanning haplotype polymorphisms common in the African American population bind MHC-II proteins significantly more than a negative control, thus providing a mechanistic explanation of the phenomenon in this population.

## Introduction

The bleeding disorder hemophilia-A (HA) is treated with infusions of plasma-derived- or recombinant (r)-Factor VIII (FVIII) [1]. The development of anti-drug antibodies that inhibit FVIII function, which occurs in approximately 20% of patients overall, is currently the most important impediment to the successful management of this chronic disease [2]. The development of inhibitory antibodies against FVIII is a complex process that involves both product- and patient-related factors [3], [4]. Genetic data could help understand why some individuals develop inhibitory antibodies, while others do not, even when an essentially identical recombinant FVIII protein-drug is infused in all patients. Our recent work suggests that non-synonymous Single Nucleotide Polymorphisms (ns-SNPs) in the *F8* gene of some HA patients could be a pharmacogenetic risk-factor for developing inhibitory anti-drug antibodies [5].

At least six variants of the *F8* gene (designated H1 through H6) are found in normal individuals who do not suffer from HA, but only two (H1 and H2) match the recombinant FVIII products used clinically. While H1 and H2 were found in all racial groups studied, H3, H4 and H5 have been found so far only in Americans of black African descent, and H6 was found in a few persons of Chinese descent [6]. In 78 black patients with HA, 24% had an H3 or H4 background haplotype and the prevalence of inhibitory anti-drug antibodies was higher among patients with the H3 or H4 haplotypes than among patients with haplotype H1 or H2 (odds ratio, 3.6; 95% confidence interval, 1.1 to 12.3; P-value = 0.04) [5]. These results suggested that a mismatch in the sequences of the endogenous (albeit non-functional) FVIII synthesized by the patient and the infused FVIII protein drug could be the underlying cause for the high incidence of inhibitory anti-drug antibodies among black patients. This hypothesis has, however, not been experimentally verified and the underlying mechanism for this clinical observation is poorly understood.

Early (though indirect) evidence that CD4+ T-cells play a central role in the development of inhibitory antibodies to FVIII came from the observation that inhibitory anti-drug antibodies spontaneously disappeared in conjunction with an HIV-associated decline in CD4+ counts [7]. Subsequently, it was demonstrated that it is possible to prevent anti-FVIII antibodies in hemophiliac mice by blocking co-stimulatory signals [8] indicating that CD4+ cells are critical. A CD4+ T-cell response to an exogenous protein requires that peptides derived from the therapeutic protein are presented by MHC-II alleles. Moreover, as individuals are tolerized to self proteins, it is necessary that these peptides be recognized as foreign. This is consistent with clinical studies which indicate that there is an association between the nature of the mutation in a patient's *F8* gene and the frequency with which inhibitory anti-drug antibodies are developed. For example, while <5% of patients with missense mutations develop inhibitory anti-drug antibodies, >60% of patients with the deletion of one or more exons do so [9]. However, even HA patients with missense mutations can have a high risk of developing inhibitory anti-drug antibodies if the mutation occurs at certain locations. For example, inhibitory anti-drug antibodies are frequently associated with the mutation R2150H in HA patients [10]. Saint-Remy and coworkers characterized FVIII-specific CD4+ T-cell clones derived from such a patient [11] and demonstrated that all T-cell clones recognized synthetic peptides encompassing R2150. Interestingly none of the T-cell clones recognized recombinant peptide with H2150, demonstrating that T-cell response was directed only at the wild-type sequence. This study is consistent with other studies showing that the anti-FVIII antibodies recognize the wild type (foreign) but not the mutant (self) protein [12]–[15]. Similarly, a more recent detailed study carried out on two unrelated patients with the R593C mutation and the MHC-II, DRB1*11:01 genotype [16] showed that wild type peptides which contain the missense site, bound to the MHC-II protein with physiologically relevant affinities. Moreover, in this study the peptide-MHC-II binding algorithm ProPred [17] was used to identify binding peptides and synthetic peptides predicted to bind with the highest affinity were tested in a binding assay using recombinant MHC-II DRB1*11:01. These reports lay the groundwork for the current study because although ns-SNPs alone do not cause HA, the amino-acid change due to an ns-SNP in the endogenous FVIII of patients, are analogous to missense mutations and can potentially result in T-cell epitopes.

The current study is designed to test the hypothesis that some polymorphisms in the endogenous *F8* gene of HA patients are risk-factors for the development of anti-drug antibodies to recombinant-FVIII used as a drug (see above and [5]). A necessary, though insufficient, condition for development of such inhibitory anti-drug antibodies is that wild type peptides, i.e. those that have the sequence of the FVIII protein therapeutic at the locations where polymorphisms occur, would be T-cell epitopes. Identification of MHC-II epitopes is an important task in basic immunological research with considerable practical value. Peptide-MHC-II prediction algorithms, if accurate enough, can replace time consuming and expensive experimental approaches. Therefore, a large number of computational methods to predict MHC-II epitopes have been developed in recent years (for review see [18]). In addition, so-called “meta” approaches have been devised, combining the results of individual methods to improve the predictive performance [19], [20].

Due to the large number of overlapping peptides that could be tested as epitopes as well as the diversity of MHC-II variants in the population it is not possible to experimentally test all possible peptide-MHC-II combinations. On the other hand, the predictive performance of peptide-MHC-II binding algorithms is, often, not good enough to justify a purely computational approach [18]. We therefore selected a few wild type peptides from regions of common polymorphisms and experimentally determined their affinities to six common MHC-II variants. These results were transformed into binding promiscuity scores that suggested that at least some polymorphic regions of the FVIII protein could yield T-cell epitopes. Furthermore, we used the complete dataset of experimentally determined “positive” and “negative” binders to re-validate two leading peptide-MHC-II binding algorithms for each of the six MHC variants; using a computational method allowed us to include many more peptide-MHC-II binding estimations per each polymorphism, significantly increasing the power of the statistical analysis. Together, the experimental and computational results support the hypothesis that peptides with the sequence of the therapeutic FVIII protein at locations where polymorphisms occur, constitute potential T-cell epitopes and thus could potentially explain the higher prevalence of inhibitory anti-drug antibodies in African American HA patients which is observed in the clinic.

## Results

### Peptide-MHC binding and stability are markers for development of anti-FVIII antibodies

**Figure 1:**
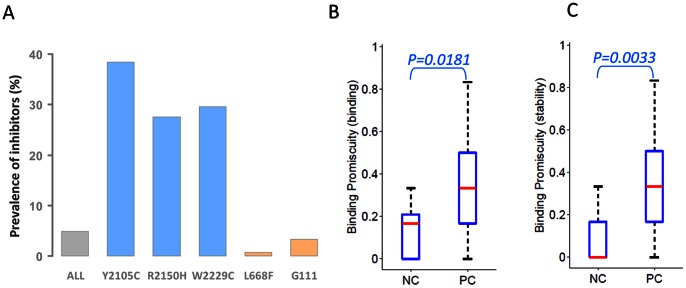
(A) Prevalence of inhibitory anti-drug antibodies in HA patients with different missense mutations in the endogenous FVIII. Graph depicts overall prevalence of inhibitory anti-drug antibodies in all HA patients with missense mutations (grey) and patients with the mutations Y2105C, R2150H, W2229C (blue) as well as L668F and G111R (tan). (**B**) Peptide-MHC-II-binding promiscuity scores. Bar and whisker plots show the binding promiscuity score, i.e. the fraction of peptide-MHC-II complexes with a binding score ≥15%, for two groups of wild type peptides, viz. positive-control (PC) and negative-control (NC) peptides. On each box, the central red mark is the median, the edges of the box are the 25^th^ and 75^th^ percentiles and the whiskers extend to the most extreme data-points. The binding scores of 16 positive control-wild type peptides and 9 negative-control wild type peptides were determined for 6 MHC-II molecules, DRB1*03:01, DRB1*04:01, DRB1*07:01, DRB1*11:01, DRB1*15:01 and DRB1*15:03. The fraction of “binders” for the positive-control group (N = 96) was significantly greater than the fraction of “binders” in the negative-control group (N = 54); one-sided MWW P-value = 0.018. (**C**) Stability- based peptide-MHC-II promiscuity scores. Bar and whisker plots show the stability-based promiscuity scores, i.e., fraction of peptide-MHC-II complexes with stability score ≥6, for two groups of wild type peptides and 6 MHC-II proteins, as depicted in (**A**). The fraction of stable complexes for the positive-control group (N = 96) was significantly greater than the fraction of “binders” in the negative-control group (N = 54); one-sided MWW P-value = 0.003.

There are over 2500 reports describing 898 unique missense mutations in HA patients (for comprehensive data base see http://hadb.org.uk/), of which very few (4.9%) developed inhibitory anti-drug antibodies. However, inhibitory anti-drug antibodies are detected in approximately 30–40% of patients with three specific missense mutations: Y205C, R2150H and W2229C. In addition, several wild type peptides from regions of the FVIII protein where these mutations occur have been extensively characterized and shown to be potent and promiscuous T-cell epitopes (see introduction, Fig. 1A and [10], [11]). In this study, we use the term “wild type peptide(s)” to denote peptides which have the same sequence as the corresponding region on the H1 variant of the FVIII protein. We screened 25 wild type FVIII peptides that included T-cell epitopes from these three regions as well as peptides from regions of the protein where missense mutations are not associated with inhibitory anti-drug antibodies (Fig. 1A). Thus, the former constituted a set of positive-control peptides and the latter negative-control peptides for evaluating peptide-MHC binding and stability as markers for the development of anti-drug antibodies. The binding and stability of each peptide was determined for six MHC-II proteins, viz. DRB1*03:01, DRB1*04:01, DRB1*07:01, DRB1*11:01, DRB1*15:01 and DRB1*15:03 in a REVEAL Assay. The binding of each peptide to an MHC-II variant was expressed as a “binding score” on a scale of 0–100; the score of each wild type FVIII peptide being relative to a unique positive control peptide, which is a known T-cell epitope for that MHC-II allele. The stability of each MHC-II-peptide complex was expressed as the normalized product of the half-life of the peptide-MHC-II complex and the binding score (see Materials and methods). In addition, we computed, for each peptide, a “binding promiscuity score” defined as the fraction of MHC-II variants that bind (binding score ≥15) or form a stable complex (stability score >6) with that peptide. The median binding promiscuity scores for positive-control wild type peptides are significantly higher than those for negative-control wild type peptides when using either peptide-MHC affinity (Fig. 1B) or stability (Fig. 1C) as a marker (one-sided Mann-Whitney-Wilcoxon (MWW) P-values = 0.018 and 0.003, respectively).

### Wild type FVIII peptides in regions of ns-SNPs bind to and form stable complexes with MHC-II alleles

**Figure 2:**
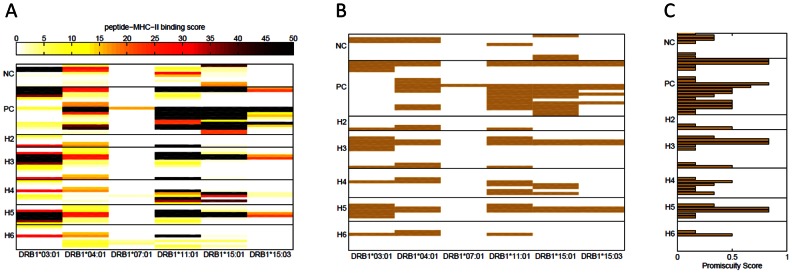
(A) Binding scores of FVIII wild type peptides. Binding scores in a REVEAL assay were measured for the synthetic wild type peptides depicted in bold in Table 1. Each row represents a peptide and each column a MHC-II allele (for raw data see Table S1). The peptides are grouped together based on the position in the FVIII gene where the haplotypes H2 to H6 occur. Overlapping peptides (4 or 5) were used around the position of each ns-SNP. Positive-control (PC) peptides are known T-cell epitopes of FVIII and negative control (NC) peptides are from regions of the FVIII protein where no T-cell epitopes have been identified. The scale for the binding score is shown at the top of the figure; dark colors represent peptides that bind with high affinity to the MHC-II allele. (**B**) FVIII high affinity binders. Wild type peptides with binding score ≥15% are shown and considered potential T-cell epitopes for each of the six MHC-II alleles. (**C**) *Binding promiscuity scores for the wild type FVIII peptides*. The binding promiscuity score for each peptide, defined as the fraction of MHC-II variants the peptide binds with a score ≥15%, is shown.

The endogenous FVIII sequences of patients with missense mutations and ns-SNPs differ from the sequence of an infused FVIII protein-therapeutic only at the locations where the mutations or ns-SNPs occur. As these patients are tolerized to the mutant or polymorphic form of the protein, the wild-type sequence of the infused FVIII might be considered “foreign” by their immune system. Using historical clinical and immunological data of FVIII missense mutations (see http://hadb.org.uk/, references therein and [9]–[12], [15]), the analyses shown in Fig. 1 suggest that wild type peptide-MHC binding and stability are both markers for identifying those peptides that are foreign to the patient and could potentially be T-cell epitopes and thus trigger the development of anti-drug antibodies. Consequently, a similar analysis should be predictive of the potential of wild type peptides that correspond to the polymorphic regions of FVIII to elicit anti-drug antibodies. We screened the binding of 30 FVIII-specific wild type peptides to the 6 MHC-II alleles listed above. These 30 peptides (Table 1) include at least 4 overlapping peptides from each location on the FVIII protein where common polymorphisms occur. Binding scores for these peptides (rows) to each of the six MHC-II proteins (columns) are shown as a heat map in Fig. 2A (binding scores are also listed in Table S1); for comparison, the binding scores of positive-control and negative-control peptides characterized in Fig. 1 are shown as well. As ns-SNPs do not always occur individually but as haplotypes, in the heat map, sets of wild type peptides are grouped together as the haplotypes H2 to H6 described previously [5], [6]. The heat map (Fig. 2A) shows that at least one peptide from each of the polymorphic regions of FVIII binds to several MHC-II variants with higher affinities than the negative-control wild type peptides. Moreover many of these peptides exhibit affinities comparable to or higher than the positive-control peptides. Positive binders identified using the same criterion used in Fig.1B (binding score ≥15%) are depicted in a binary plot (Fig. 2B). As expected, only a few binders were identified in the group of negative-control peptides while numerous binders were identified among the positive-control and test-peptides. Moreover, it is clear that even peptides that are potential T-cell epitopes do not bind to all MHC variants. Thus, to estimate the propensity of each peptide to initiate an immune response in a population we calculated the fraction of MHC-II proteins each peptide binds to (with a binding score ≥15%). Fig. 2C shows that several positive-control peptides and wild type peptides from the H3, H4 and H5 regions of FVIII bind to ≥50% (and up to 83%) of the MHC-II alleles used in this study. In contrast, the negative-control wild type peptides bind to ≤33% of MHC-II alleles.

**Table 1:** Overlapping 15 mer wild type FVIII peptides from regions 14 amino-acids upstream and downstream of the position where the amino-acid change(s) occurs due to the polymorphisms.

Haplotype	ns-SNPs	Peptides
H2	D1241E	**LFLLSTRQNVEGSYD**
		FLLSTRQNVEGSYDG
		LLSTRQNVEGSYDGA
		**LSTRQNVEGSYDGAY**
		STRQNVEGSYDGAYA
		TRQNVEGSYDGAYAP
		**RQNVEGSYDGAYAPV**
		QNVEGSYDGAYAPVL
		NVEGSYDGAYAPVLQ
		VEGSYDGAYAPVLQD
		EGSYDGAYAPVLQDF
		**GSYDGAYAPVLQDFR**
		SYDGAYAPVLQDFRS
		YDGAYAPVLQDFRSL
		**DGAYAPVLQDFRSLN**
H3	D1241E	**LFLLSTRQNVEGSYD**
		FLLSTRQNVEGSYDG
		LLSTRQNVEGSYDGA
		**LSTRQNVEGSYDGAY**
		STRQNVEGSYDGAYA
		TRQNVEGSYDGAYAP
		**RQNVEGSYDGAYAPV**
		QNVEGSYDGAYAPVL
		NVEGSYDGAYAPVLQ
		VEGSYDGAYAPVLQD
		EGSYDGAYAPVLQDF
		**GSYDGAYAPVLQDFR**
		SYDGAYAPVLQDFRS
		YDGAYAPVLQDFRSL
		**DGAYAPVLQDFRSLN**
	M2238V	**NNPKEWLQVDFQKTM**
		NPKEWLQVDFQKTMK
		**PKEWLQVDFQKTMKV**
		KEWLQVDFQKTMKVT
		**EWLQVDFQKTMKVTG**
		WLQVDFQKTMKVTGV
		**LQVDFQKTMKVTGVT**
		QVDFQKTMKVTGVTT
		VDFQKTMKVTGVTTQ
		DFQKTMKVTGVTTQG
		FQKTMKVTGVTTQGV
		**QKTMKVTGVTTQGVK**
		KTMKVTGVTTQGVKS
		TMKVTGVTTQGVKSL
		**MKVTGVTTQGVKSLL**
H4	D1241E	**LFLLSTRQNVEGSYD**
		FLLSTRQNVEGSYDG
		LLSTRQNVEGSYDGA
		**LSTRQNVEGSYDGAY**
		STRQNVEGSYDGAYA
		TRQNVEGSYDGAYAP
		**RQNVEGSYDGAYAPV**
		QNVEGSYDGAYAPVL
		NVEGSYDGAYAPVLQ
		VEGSYDGAYAPVLQD
		EGSYDGAYAPVLQDF
		**GSYDGAYAPVLQDFR**
		SYDGAYAPVLQDFRS
		YDGAYAPVLQDFRSL
		**DGAYAPVLQDFRSLN**
	R484H	**SRPYNIYPHGITDVR**
		RPYNIYPHGITDVRP
		PYNIYPHGITDVRPL
		**YNIYPHGITDVRPLY**
		NIYPHGITDVRPLYS
		IYPHGITDVRPLYSR
		**YPHGITDVRPLYSRR**
		PHGITDVRPLYSRRL
		HGITDVRPLYSRRLP
		**GITDVRPLYSRRLPK**
		ITDVRPLYSRRLPKG
		TDVRPLYSRRLPKGV
		**DVRPLYSRRLPKGVK**
		VRPLYSRRLPKGVKH
		RPLYSRRLPKGVKHL
H5	M2238V	**NNPKEWLQVDFQKTM**
		NPKEWLQVDFQKTMK
		**PKEWLQVDFQKTMKV**
		KEWLQVDFQKTMKVT
		**EWLQVDFQKTMKVTG**
		WLQVDFQKTMKVTGV
		**LQVDFQKTMKVTGVT**
		QVDFQKTMKVTGVTT
		VDFQKTMKVTGVTTQ
		DFQKTMKVTGVTTQG
		FQKTMKVTGVTTQGV
		**QKTMKVTGVTTQGVK**
		KTMKVTGVTTQGVKS
		TMKVTGVTTQGVKSL
		**MKVTGVTTQGVKSLL**
H6	D1241E	**LFLLSTRQNVEGSYD**
		FLLSTRQNVEGSYDG
		LLSTRQNVEGSYDGA
		**LSTRQNVEGSYDGAY**
		STRQNVEGSYDGAYA
		TRQNVEGSYDGAYAP
		**RQNVEGSYDGAYAPV**
		QNVEGSYDGAYAPVL
		NVEGSYDGAYAPVLQ
		VEGSYDGAYAPVLQD
		EGSYDGAYAPVLQDF
		**GSYDGAYAPVLQDFR**
		SYDGAYAPVLQDFRS
		YDGAYAPVLQDFRSL
		**DGAYAPVLQDFRSLN**
	R776G	**PENDIEKTDPWFAHR**
		ENDIEKTDPWFAHRT
		NDIEKTDPWFAHRTP
		**DIEKTDPWFAHRTPM**
		IEKTDPWFAHRTPMP
		EKTDPWFAHRTPMPK
		**KTDPWFAHRTPMPKI**
		TDPWFAHRTPMPKIQ
		DPWFAHRTPMPKIQN
		**PWFAHRTPMPKIQNV**
		WFAHRTPMPKIQNVS
		FAHRTPMPKIQNVSS
		**AHRTPMPKIQNVSSS**
		HRTPMPKIQNVSSSD
		RTPMPKIQNVSSSDL

Peptides for which binding affinity was experimentally measured are shown in bold.

**Figure 3:**
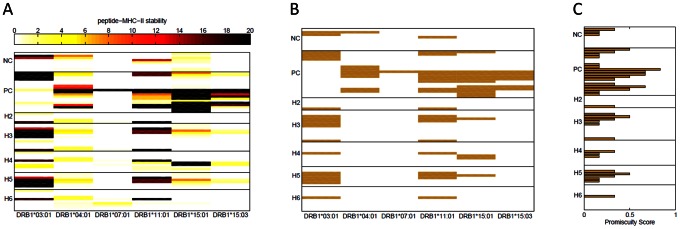
(A) Stability of FVIII peptide-MHC-II complexes. Half-life times for the synthetic wild type peptides depicted in bold in Table 1 with each peptide-MHC-II were determined and normalized to a stability score (see Materials and Methods for details). Each row represents a peptide and each column a MHC-II allele. The peptide groupings as well as the negative-control (NC) and positive-control (PC) wild type peptides are the same as those shown in Fig. 1A. (**B**) Stable FVIII peptide-MHC-II complexes. Peptide-MHC-II complexes with stability score ≥6 are shown and considered stable. (**C**) Binding promiscuity scores for the wild type FVIII peptides based on stability. The fraction of MHC-II alleles each peptide binds to with a stability score ≥6 is shown.

In addition to peptide-MHC-II binding *per se*, the stability of such complexes has been shown to be an important parameter for predicting the eventual immune response [21]–[25]. A heat map depicting the stability index for negative-control, positive-control- and test-peptides (Fig. 3A) shows that more stable peptide-MHC-II complexes are generated between MHC-II proteins and the positive-control and test-peptides than between MHC-II proteins and the negative-control peptides. The binary-plot (Fig. 3B) shows the peptides that bind with a stability score ≥6. As expected, the stability score is more stringent than the binding score and thus several peptides form stable complexes with fewer MHC-II proteins (compare Figs. 2C and 3C).

### Evaluation of prediction tools using the FVIII peptide-MHC-II experimental data

**Figure 4:**
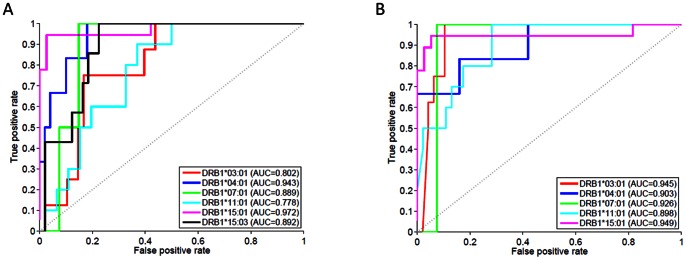
Performance of computational MHC class II binding prediction methods. ROC curves comparing measured and predicted binding affinities using NetMHCIIpan-2.1 (**A**) and the Consensus method (**B**) for 54 FVIII wild type peptides to the following MHC-II alleles: DRB1*03:01 (red), DRB1*04:01 (blue), DRB1*07:01 (green), DRB1*11:01 (cyan), DRB1*15:01 (magenta) and DRB1*15:03 (black). Area under the ROC curve values are depicted on the figure.

The experimental approaches described in Figs. 1–3 utilized six MHC-II variants. Moreover, rather than synthesizing all overlapping 15 mer wild type peptides around the location of each ns-SNP we sampled only 4–5 of the 15 peptides that could be generated. Binding of a complete coverage of all overlapping wild type peptides (Table 1) and a greater diversity of MHC-II variants can be evaluated using a computational approach. Publicly available prediction tools have been previously evaluated [18], [19] and shown to perform well in some cases but poorly in others. We therefore evaluated the predictive performance of two computational tools, NetMHCIIpan-2.1 [26] and the Consensus method [19] using the experimentally determined affinities of 54 FVIII-wild type peptides for the six MHC-II alleles described above. Although this is a much smaller data-set than previous evaluations our purpose was to select a method that best fits our FVIII-based experimental data. Figs. 4 A,B show the prediction performance of the Consensus and NetMHCIIpan-2.1 methods in terms of area under ROC curve (AUC). Both methods show good predictive performance (with a slight advantage to the Consensus method with respect to the AUC): AUCs ranged from 0.778 to 0.972 and 0.898 to 0.949 for the NetMHCIIpan-2.1 and Consensus methods respectively. However, as NetMHCIIpan-2.1 predicts binding for a larger set of MHC-II variants, thus covering a larger portion of the population, we chose this method in our analyses.

### Expanding the range of wild type peptides and MHC-II alleles evaluated using a computational approach

**Figure 5:**
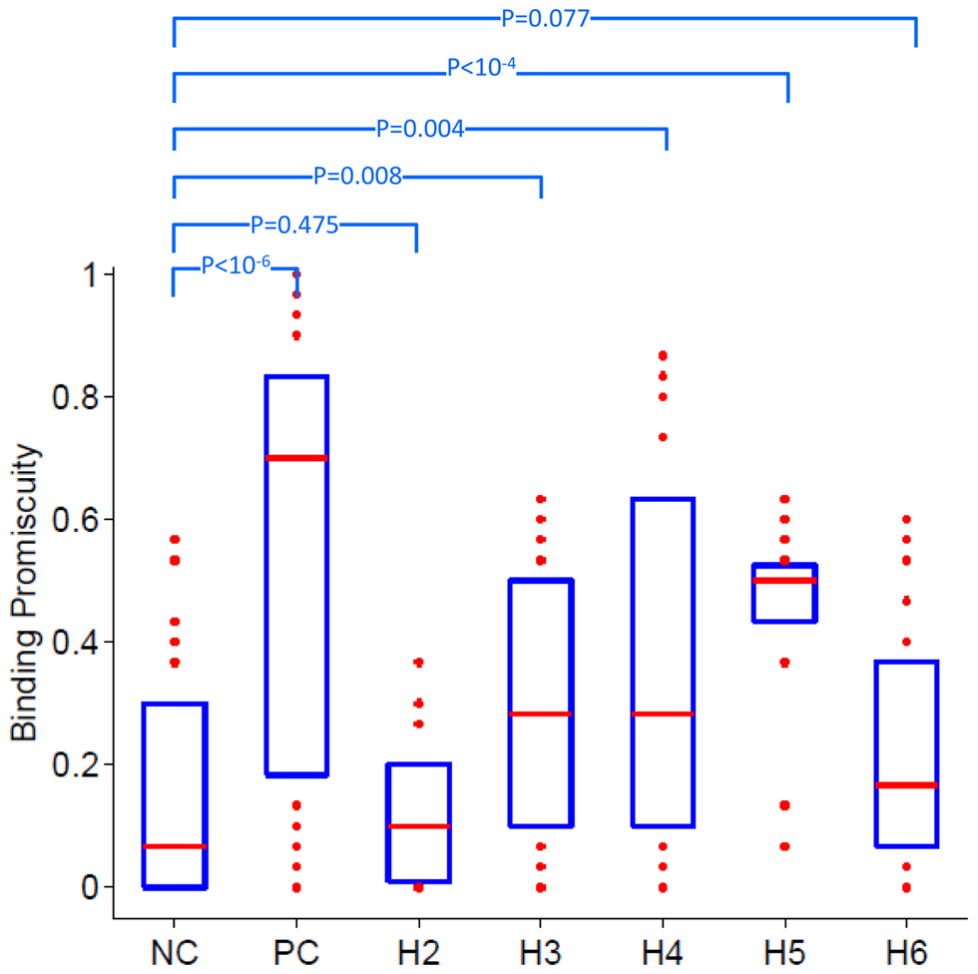
Statistics of predicted binding promiscuity scores for haplotypes H2 to H6. We calculated the binding promiscuity scores, based on NetMHCIIpan-2.1 predicted binding affinities, for 30 HLA-DRB1 alleles to all 15 mer overlapping wild type peptides that incorporate the location of each ns-SNP. The numbers of wild type peptides in each group are: negative-control (NP), 30; positive-control (PC), 45; H2, 15; H3, 30; H4, 30; H5, 15 and H6, 30. Each bar and whisker plot depicts the binding promiscuity score for all peptides in the group; i.e. the fraction of the 30 MHC-II variants that peptide binds to. On each box, the central red mark is the median, the edges of the box are the 25th and 75th percentiles, and data-points outside this range are plotted individually. Median binding promiscuity scores for haplotype H3 to H5 are significantly greater than those of the negative-control (one-sided MWW P-values depicted on figure).

Using the computational tool NetMHCIIpan-2.1 we predicted the affinity of all overlapping wild type peptides from polymorphic regions of FVIII (all peptides in Table 1) to 30 MHC-II alleles. Together, these MHC-II alleles occur in at least 90% of the North American, African or World populations. Similarly, the negative-control and positive-control peptides used in this computational analysis included all overlapping wild type peptides in the regions of FVIII depicted in Fig. 1. Following previous studies, we considered peptides with predicted affinity ≤500 nM as binders [27]; as described above, binding promiscuity score was defined as the fraction of MHC-II variants each peptide binds to. A box and whisker plot (Fig. 5) shows that the median binding promiscuity score for positive-control FVIII peptides is significantly greater than that for negative-control FVIII peptides (one-sided MWW, P-value<10^−6^). Interestingly, wild type peptides from the polymorphic regions of FVIII exhibit a range of median binding promiscuity scores. For example the median binding promiscuity score of all H2 and H6 wild type peptides is not significantly different from that of negative-control peptides while wild type peptides from locations where the H3, H4 and H5 polymorphisms occur have significantly higher scores than negative-control wild type peptides (one-sided MWW P-values of 0.008, 0.004 and <10^−4^ respectively).

### Wild type FVIII peptides in regions of ns-SNPs bind with high affinity to MHC-II alleles that occur frequently in African populations

**Figure 6:**
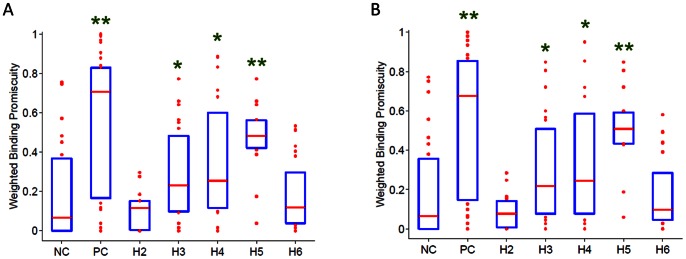
Binding promiscuity scores weighted for the frequencies of the different MHC-II variants in the North American and African populations. Weighted binding promiscuity scores, based on NetMHCIIpan-2.1 predicted binding affinities for 30 HLA-DRB1 alleles to all 15 mer overlapping wild type peptides that incorporate the location of each ns-SNP were calculated. For each peptide, the weighted binding promiscuity score is the sum of frequencies in the North American (**A**) or African (**B**) populations of the MHC-II to which it binds (affinity ≤500 nM), normalized by the total cumulative frequency of all 30 MHC-II variants used in the analysis. Each bar and whisker plot depicts the appropriately weighted binding promiscuity score for all wild type peptides in that group; for interpretation of box and whisker plots see Fig. 5. The hypothesis that the median of the weighted binding promiscuity scores for each group is significantly greater than that of the negative-control was tested using a one-sided MWW test; groups with P-values<0.05 (*) and <10^−3^ (**) are shown above the bars. Median binding promiscuity score statistics for haplotype H3-H5 are significantly greater than that of the negative-control for both (**A**) and (**B**).

In the data presented above, we computed an (un-weighted) binding promiscuity score to estimate the prevalence of an immunogenic response to a specific peptide within the population (Figs. 1C & 5). Next, we analyze a binding promiscuity score weighted for the frequency with which the different MHC-II alleles occur in the North American population; Fig. 6A compares the median of these weighted binding promiscuity scores for positive-control-, negative-control-, and test-peptides (all of which carry the wild type sequence; Fig. S1B shows the corresponding scores per each ns-SNP position). Although the specific median binding promiscuity scores for the different groups of peptides vary, overall the pattern remains the same, i.e. wild type peptides from the H2 and H6 regions of FVIII do not have significantly higher median binding promiscuity scores than negative-control peptides while wild type peptides from the H3, H4 and H5 locations have significantly higher binding promiscuity scores.

**Figure 7:**
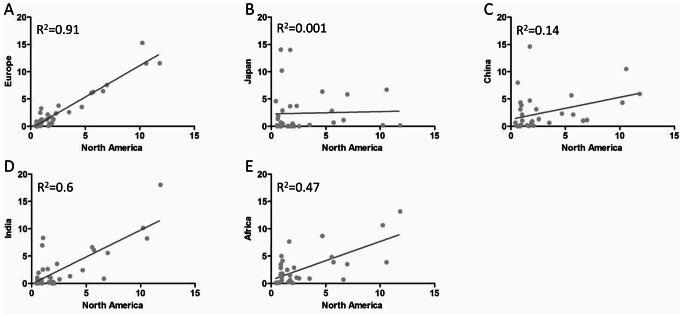
Diversity of MHC-II distributions. Correlation of the distribution of 30 MHC-II DRB1* alleles in European (**A**), Japanese (**B**), Chinese (**C**), Indian (**D**) and African (**E**) populations and the distribution of the same MHC-II DRB1* alleles in the North American population. Individual MHC-II DRB1* alleles occur at frequencies that range from <1% to almost 15% (see any X- or Y-axis). The distribution of these alleles can be comparable between some populations, e.g. the North American and European populations (**A**, correlation coefficient, 0.95) or very different, e.g. North American and Japanese population (**B**, correlation coefficient, 0.03). The primary source for this data is the Allele Frequency Net Database (www.allelefrequencies.net). See Materials and Methods for additional details.

It is also important to note that polymorphisms in the *F8* gene exhibit racial differences. For example, studies have shown that the haplotypes H3 and H4 in FVIII occur very infrequently (if at all) in individuals of Caucasian descent while they occur in about a third of individuals of Black African descent. Moreover the distribution of MHC-II alleles is also different between different populations (Fig. 7). Thus, the distribution of MHC-II alleles in the African population may be more appropriate than the distribution in the North American population as a whole for studying the higher incidence of anti-drug antibodies to FVIII in African-American HA patients than in Caucasian patients. As shown in Fig. 6B, wild type peptides that occur at the location of the H3, H4 and H5 variants have significantly higher weighted binding promiscuity scores than the negative control peptides. Breakdown for individual ns-SNP positions (Fig. S1C) suggests that the drivers for the significantly higher binding promiscuity scores of wild-type haplotypes H3 and H4 are peptides that incorporate M2238 and R484 respectively.

## Discussion

The prevalence of anti-drug antibodies in African American HA patients is twice that observed in white patients of Caucasian descent [5]. Recent clinical studies from one of our laboratories determined that the endogenous FVIII protein is much more polymorphic in black individuals than in white individuals [5]. Moreover, FVIII-protein-therapeutics in the market are more likely to be matched, vis-à-vis the sequence, to the endogenous FVIII of a Caucasian patient than an African American patient. Taking these observations into account we reported that polymorphisms in the endogenous *F8* gene are a risk factor for developing inhibitory anti-drug antibodies (odds ratio, 3.6; 95% confidence interval, 1.1 to 12.3; P-value = 0.04). The clinical findings suggest that wild type peptide sequences that correspond to the regions of polymorphisms may constitute T-cell epitopes and trigger T-cell activation. In this study we use experimental and computational methods to evaluate these putative T-cell epitopes as biomarkers for the development of anti-drug antibodies.

In the first, experimental part of the study, we measured peptide-MHC-II affinity and stability and use it as a surrogate marker for the development of anti-drug antibodies. To assess our method's capability to predict a clinical manifestation of anti-drug antibodies we used 16 positive-control peptides, i.e. well characterized T-cell epitopes from FVIII associated with the development of inhibitory anti-drug antibodies [12]–[15] and 9 FVIII-derived negative-control peptides. The former have significantly higher median binding promiscuity scores than the latter (one-sided MWW P-values = 0.018 and 0.003 for binding promiscuity scores based on peptide-MHC-II affinity and stability, respectively) (Fig. 1B, C).

Assessing the risk of developing anti-drug antibodies is a two-step process. The first step involves considering variables that are anticipated to affect the likelihood of developing anti-drug antibodies and the clinical consequences if such antibodies are generated. The second step is determining the risk associated with the development of anti-drug antibodies as a function of the severity of the clinical consequence and the probability of that occurrence in a patient population. We devised a binding promiscuity score based on these principles. We first determined whether or not a peptide was a “binder” for each MHC-II variant based on a predetermined cut-point (Figs. 2B & 3B). This is a surrogate marker for developing anti-drug antibodies and extensive clinical experience with HA demonstrates that the consequences can be very severe [1]–[3]. Secondly we calculated the binding promiscuity score as the fraction of MHC-II molecules each peptide bound to based on the cut-point (Figs. 2C & 3C). Assuming that the MHC-II variants used in the study are representative of those in the population, this score is a measure of the risk of developing anti-drug antibodies that is associated with that haplotype in the patient population. The caveat is that the predictive power of such a binding promiscuity score is limited by the coverage of the population with respect to the number of MHC-II proteins studied and their frequency of occurrence in the population. For example the 6 MHC-II proteins used in this study occur in 46.5% and 40.8% of the North American and African populations respectively.

Thus, although our experimental studies suggest that several wild type peptides from the H3, H4 and H5 regions of FVIII are plausible candidates for T-cell epitopes in a patient population, the data set is limited by both the number of foreign peptides and MHC-II variants tested. Cost constrains and the availability of recombinant MHC-II variants limit the generation of complete data sets (i.e., all possible overlapping wild type peptides and MHC-II protein combinations). It is for reasons such as these that considerable effort has been expended in the last decades to develop computational methods to predict MHC-peptide binding [18], [19]. In general, computational tools to predict peptide-MHC binding perform better for class-I proteins than for class-II molecules. Recent years, however, have seen a significant improvement in the prediction accuracy of class-II tools [18], [19]. In particular, methods that combine the results of several tools (e.g., the Consensus algorithm; [19]) as well as pan-specific algorithms, which can predict binding for proteins with no experimental binding data (e.g., NetMHCIIpan; [26]) have been devised.

We evaluated the performance of the Consensus [19] and NetMHCIIpan-2.1 [26] prediction methods for the 54 FVIII wild type peptides used in this study. Both methods showed good performance for all six MHC-II alleles with AUC values in the range of 0.778 to 0.972, which is consistent with previous reports [19].

As the NetMHCIIpan-2.1 method provides prediction for a larger set of MHC-II proteins, we used it to estimate the binding of all overlapping 15 mer wild type peptides in the polymorphic, as well as control, regions of FVIII to 30 MHC-II variants. The cumulative frequency of these MHC proteins is 97.7% and 90.5% in the North American and African populations, respectively. Compared to the negative-control peptides, the median binding promiscuity scores for the positive-control peptides and test-peptides from regions H3, H4 and H5 are significantly higher (Fig. 5), consistent with the experimental conclusions that polymorphic regions of FVIII are likely to generate positive T-cell epitopes (see above and Figs. 2 and 3).

Expanding the repertoire of MHC-II variants by using computational methods provides another important advantage for this study: We can better understand what the consequences of a particular polymorphism would be in the general population as well as in specific sub-populations. In our calculations, the binding promiscuity score for each peptide is the fraction of MHC-II alleles that the peptide binds to with a higher affinity than a predetermined cut-point. Thus, if peptides from one region of the FVIII protein have median binding promiscuity scores that are significantly higher than those of another region they are likely to be more immunogenic in the population represented by the MHC variants. Furthermore, as the peptide binding is MHC restricted, the added advantage of expanding the number of MHC-II variants studied is that, the better the coverage of the population vis-à-vis MHC variants the better the risk assessment. It is important to note that the distribution of MHC-II variants is not uniform in the North American population, the different variants occur at frequencies that range from <1% to >10% (Fig. 7A). In addition there are considerable geographical differences in the distribution of the MHC-II variants (Fig. 7) which are likely maintained, to some extent, in US sub-populations originating from those geographical regions. Thus the binding promiscuity scores weighted for the frequencies of the different MHC-II variants in the population(s) of interest are more likely to provide more accurate prediction of risk for that population (Fig. 6). Moreover, such an analysis can also provide some mechanistic insights. For example, in the analysis performed in this study the promiscuity scores weighted for distribution of MHC-II alleles in the North American and African populations are comparable. This observation could suggest the hypothesis that higher prevalence of inhibitory anti-drug antibodies in the African-American sub-population may be mediated through recognition of the MHC-II-peptide complex by the T-cell receptor and not restriction in MHC-II binding. A comprehensive experimental determination of all possible peptide-MHC-II binding affinities required to generate such a weighted binding promiscuity score would be prohibitive in cost and present a considerable technical challenge. Despite using a large repertoire of MHC-II variants, the analysis of peptide-MHC-II interactions in this study was limited to DR molecules. A previous study demonstrated that peptides that contained FVIII residues I2144-T2161 strongly stimulated T-cell clones. However this response was completely abrogated by a monoclonal antibody to MHC-II DR molecules but not by monoclonal antibodies to MHC class II DP and DQ molecules [11]. Nonetheless it has not been conclusively established that the MHC class II restriction is mediated by DR molecules. Thus future studies involving MHC-II DP and DQ molecules would be useful.

In conclusion, this study provides proof-of-principle that predictive algorithms for peptide-MHC-II binding can be used in conjunction with limited experimental studies to obtain mechanistic insights that have potential clinical importance. Our analyses suggest that: (i) peptide-MHC-II affinity could be used as a predictive marker for clinical manifestations of anti-drug antibodies of FVIII molecules used as protein-therapeutics (Figs. 1B,C, 5 and 6); (ii) Publicly available peptide-MHC-II prediction methods show good predictive performance with respect to our experimentally determined affinity (Fig. 4); (iii) Both experimental and computational affinities of peptide-MHC-II binding suggest that wild type peptides from some polymorphic regions of the FVIII proteins can generate strong T-cell epitopes (Figs. 2, 3, 5 and 6); and (iv) The HLA-restriction of these potential T-cell epitopes suggests extensive penetration in the North American population as well as among African Americans (who constitute the more relevant population, as *F8* polymorphisms occur predominantly in this group) (Fig. 6).

## Materials and Methods

### Cell-free REVEAL class II binding assay

We used a proprietary high throughput, REVEAL binding assay (ProImmune, www.proimmune.com) to estimate the binding of individual test peptides to the following MHC-II variants: DRB1*03:01, DRB1*04:01, DRB1*07:01, DRB1*11:01, DRB1*15:01 and DRB1*15:03 [28]. Detection of a peptide binding to each MHC-II protein is based on the presence or absence of the native conformation of the MHC-peptide complex. The peptide-MHC-II complex is detected by an increase in a fluorescence signal due to the binding of a specific monoclonal antibody. Each test-peptide is given a score relative to a positive control peptide, which is a known T-cell epitope for that MHC-II molecule. The score is reported quantitatively as a percentage of the signal generated by the test peptide compared with the positive control peptide. Assay performance is evaluated by including in each set of measurements an intermediate control peptide which is known to bind the MHC-II molecule but with a lower affinity than the positive control peptide. Note that the positive control peptide used to determine the binding score is different from the positive control peptides depicted in the figures, which are known T-cell epitopes identified in the FVIII molecule.

### Cell-free REVEAL class II stability assay

We measured the half-life of each peptide-MHC-II complex using REVEAL peptide-MHC affinity assays developed by ProImmune Ltd. (Oxford, UK) [29]. On-rates of peptides were measured over a 96-hr period at 10°C. A unique positive control peptide was used for each MHC-II allele. Samples of assembling peptide-MHC complexes were taken at defined time points and snap-frozen in liquid nitrogen prior to analysis. Assembly of peptide-MHC complexes was detected using a conformational ELISA involving an anti-HLA antibody. On-rates were calculated by fitting data to a one-phase association curve: 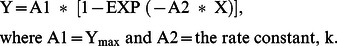
To determine the off-rates of the peptides, samples of dissociating complexes were taken at defined time points and frozen in liquid nitrogen prior to analysis. Percent denaturation of peptide-MHC complexes was also detected using a conformational ELISA. Off-rates were calculated by fitting data to a one-phase dissociation curve:
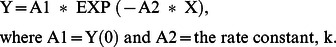
On- and off-rate half-life values (t_1/2_) were calculated from the rate constant (k) using the following equation:
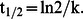



To obtain a normalized stability score, the half-life values were multiplied by the Binding Score (see above) and divided by 100. Also because the assay cannot obtain accurate half-life values above 120 hours, all values above this time-point were taken to be 120 in the calculation.

### MHC-II allele frequency data

The primary source of the MHC-II allele frequencies used in determining the weighted binding promiscuity score is the Allele Frequency Net Database (www.allelefrequencies.net). For a given geographical region all the available studies are taken and the specific HLA-allele frequencies of interest are averaged, weighting the analysis on the number of individuals in the study. Care is taken to exclude studies that are based solely on single families or anthropological studies that are based on groups accounting for small proportions of the population. Where possible, data was calculated from large-scale general population sampling studies such as donor banks, as these are assumed to be representative of the population as a whole.

### Binding promiscuity score

The binding promiscuity score of a peptide is computed using its binding score (measured or predicted) or the stability score, to a set of HLA-DRB1 alleles, and an associated cut-point. The (un-weighted) binding promiscuity score is defined as the fraction of HLA-DRB1 alleles with score greater than the predefined threshold. The weighted binding promiscuity score also considers the frequency of each HLA-DRB1 in the population of interest and is defined as the sum of the frequencies of binding HLA-DRB1s, normalized by the total cumulative frequency of the alleles in the set. In this study, the experimental binding promiscuity score is based on measurements for six HLA-DRB1s, representing 46.5% of the North American population, and binding cut-points of >15% for REVEAL binding score and >6 for REVEAL stability score. Predicted binding affinities were computed by NetMHCIIpan-2.1 [30] and are given in nM, where peptides with affinities ≤500 nM are considered binders.

### Disclaimer

The findings and conclusions in this article have not been formally disseminated by the Food and Drug Administration and should not be construed to represent any Agency determination or policy.

## Supporting Information

Figure S1 **Binding promiscuity scores for wild-type peptides from locations where ns-SNPs in the F8 gene occur.** Promiscuity scores, based on NetMHCIIpan-2.1 predicted binding affinities for 30 HLA-DRB1 alleles to all 15 mer overlapping wild type peptides that incorporate the location of each ns-SNP were calculated. The un-weighted score (**A**) as well as promiscuity scores which are weighted for the frequency of the MHC-II variants in the North American (**B**) or African (**C**) populations are depicted. Each bar and whisker plot depicts the appropriately weighted binding promiscuity score for all wild type peptides in that group; for interpretation of the plots see Fig. 5. The hypothesis that the median of the promiscuity scores for each group is significantly greater than that of the negative-control was tested using a one-sided MWW test; groups with P-values<0.05 (*) and <10^−3^ (**) are shown above the bars.
(TIF)

Table S1 **Binding scores and stability-index values for all peptide-MHC-II complexes examined in this study.**
(DOC)
