# Supplementary figures and images for: Polymorphisms in the F8 Gene and MHC-II Variants as Risk Factors for the Development of Inhibitory Anti-Factor VIII Antibodies during the Treatment of Hemophilia A: A Computational Assessment
Source: PLoS Comput Biol. 2013 May 16;9(5):e1003066. doi: 10.1371/journal.pcbi.1003066 (PMC3656107; doi:10.1371/journal.pcbi.1003066)

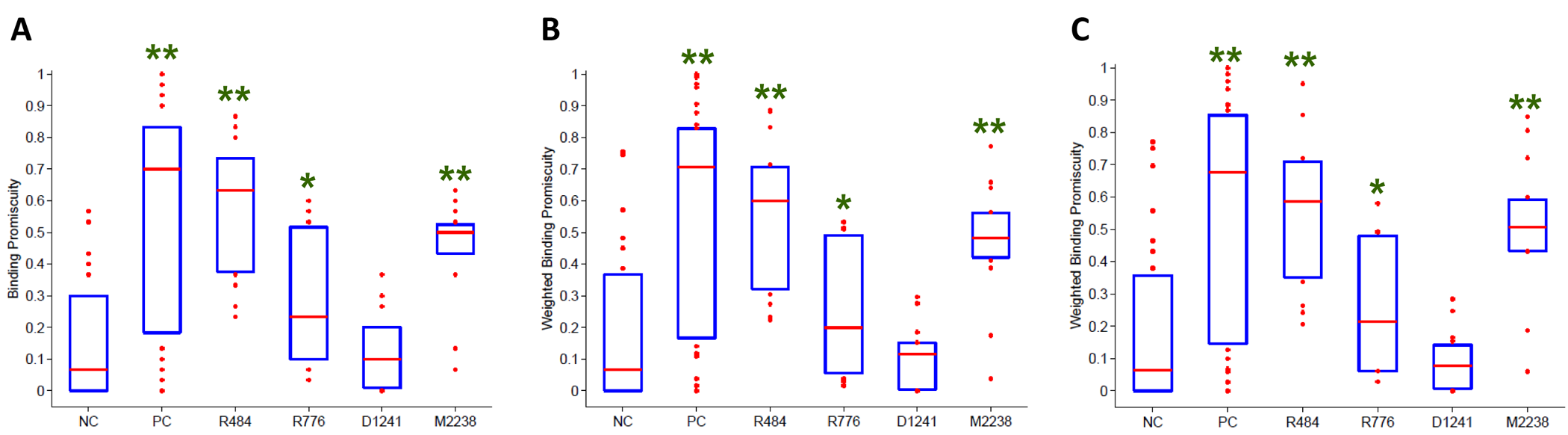

Supplement: Figure S1 — Binding promiscuity scores for wild-type peptides from locations where ns-SNPs in the F8 gene occur. Promiscuity scores, based on NetMHCIIpan-2.1 predicted binding affinities for 30 HLA-DRB1 alleles to all 15 mer overlapping wild type peptides that incorporate the location of each ns-SNP were calculated. The un-weighted score (A) as well as promiscuity scores which are weighted for the frequency of the MHC-II variants in the North American (B) or African (C) populations are depicted. Each bar and whisker plot depicts the appropriately weighted binding promiscuity score for all wild type peptides in that group; for interpretation of the plots see Fig. 5. The hypothesis that the median of the promiscuity scores for each group is significantly greater than that of the negative-control was tested using a one-sided MWW test; groups with P-values<0.05 (*) and <10−3 (**) are shown above the bars. (TIF) [file pcbi.1003066.s001.tif]
