# Supplementary material for: Polymorphisms in the F8 Gene and MHC-II Variants as Risk Factors for the Development of Inhibitory Anti-Factor VIII Antibodies during the Treatment of Hemophilia A: A Computational Assessment
Source: PLoS Comput Biol. 2013 May 16;9(5):e1003066. doi: 10.1371/journal.pcbi.1003066 (PMC3656107; doi:10.1371/journal.pcbi.1003066)
Supplement: Table S1 — Binding scores and stability-index values for all peptide-MHC-II complexes examined in this study. (DOC) [file pcbi.1003066.s002.doc]

**Table S-1**. Binding scores and stability-index values for all peptide-MHC-II complexes examined in this study.

| **DRB1*15:01** | | |
| --- | --- | --- |
| **Peptide** | **Binding Score** | **Stability Index** |
| SRPYNIYPHGITDVR | 0.1 | 0.1 |
| YNIYPHGITDVRPLY | 40.8 | 2.0 |
| YPHGITDVRPLYSRR | 0.4 | 0.5 |
| GITDVRPLYSRRLPK | 29.1 | 34.9 |
| DVRPLYSRRLPKGVK | 56.2 | 67.4 |
| PENDIEKTDPWFAHR | 0.1 | 0.1 |
| DIEKTDPWFAHRTPM | 2.6 | 0.2 |
| KTDPWFAHRTPMPKI | 0.1 | 0.1 |
| PWFAHRTPMPKIQNV | 4.7 | 0.4 |
| AHRTPMPKIQNVSSS | 0.0 | 0.1 |
| LFLLSTRQNVEGSYD | 1.0 | 0.1 |
| LSTRQNVEGSYDGAY | 0.0 | 0.0 |
| RQNVEGSYDGAYAPV | 0.0 | 0.0 |
| SYDGAYAPVLQDFRS | 0.0 | 0.0 |
| DGAYAPVLQDFRSLN | 0.0 | 0.0 |
| NNPKEWLQVDFQKTM | 0.1 | 0.2 |
| PKEWLQVDFQKTMKV | 0.4 | 0.4 |
| EWLQVDFQKTMKVTG | 59.5 | 3.8 |
| LQVDFQKTMKVTGVT | 4.4 | 0.3 |
| QKTMKVTGVTTQGVK | 0.8 | 0.5 |
| MKVTGVTTQGVKSLL | 1.4 | 0.8 |
| QKFSSLYISQFIIMY | 22.9 | 27.5 |
| FSSLYISQFIIMYSL | 23.3 | 28.0 |
| YISQFIIMYSLDGKK | 602.6 | 723.1 |
| SQFIIMYSLDGKKWQ | 1455.4 | 1746.5 |
| IIMYSLDGKKWQTYR | 1853.0 | 2223.6 |
| YSLDGKKWQTYRGNS | 11.8 | 3.0 |
| IFNPPIIARYIRLHP | 1224.5 | 1469.4 |
| NPPIIARYIRLHPTH | 4136.7 | 1374.9 |
| PIIARYIRLHPTHYS | 917.4 | 1100.9 |
| IARYIRLHPTHYSIR | 3379.6 | 4055.5 |
| YIRLHPTHYSIRSTL | 1195.7 | 1434.9 |
| RSNAWRPQVNNPKEW | 0.4 | 0.5 |
| AWRPQVNNPKEWLQV | 0.5 | 0.6 |
| PQVNNPKEWLQVDFQ | 0.1 | 0.2 |
| PKEWLQVDFQKTMKV | 0.9 | 1.1 |
| EWLQVDFQKTMKVTG | 129.0 | 8.0 |
| SGYTFKHKMVYEDTL | 1.0 | 1.2 |
| YTFKHKMVYEDTLTL | 0.9 | 1.1 |
| HKMVYEDTLTLFPFS | 2.4 | 2.9 |
| KMVYEDTLTLFPFSG | 3.0 | 0.2 |
| TLTLFPFSGETVFMS | 41.4 | 2.3 |
| VVNSLDPPLLTRYLR | 95.8 | 7.8 |
| PPLLTRYLRIHPQSW | 403.3 | 483.9 |
| PLLTRYLRIHPQSWV | 714.7 | 857.7 |
| TRYLRIHPQSWVHQI | 819.2 | 983.1 |
| RIHPQSWVHQIALRM | 8.3 | 10.0 |
| TVEDGPTKSDPRCLT | 0.0 | 0.0 |
| GPTKSDPRCLTRYYS | 63.6 | 7.2 |
| TKSDPRCLTRYYSSF | 37.9 | 45.5 |
| SDPRCLTRYYSSFVN | 237.9 | 285.5 |
| PRCLTRYYSSFVNME | 336.9 | 404.2 |
| SLHAVGVSYWKASEG | 18.1 | 1.4 |
| AVGVSYWKASEGAEY | 17.0 | 1.0 |
| SYWKASEGAEYDDQT | 0.0 | 0.0 |
| ASEGAEYDDQTSQRE | 0.0 | 0.0 |
| **DRB1*03:01** | | |
| **Peptide** | **Binding Score** | **Stability Index** |
| SRPYNIYPHGITDVR | 0.1 | 0.0 |
| YNIYPHGITDVRPLY | 0.2 | 0.0 |
| YPHGITDVRPLYSRR | 4.8 | 5.2 |
| GITDVRPLYSRRLPK | 10.8 | 3.7 |
| DVRPLYSRRLPKGVK | 0.1 | 0.2 |
| PENDIEKTDPWFAHR | 0.0 | 0.0 |
| DIEKTDPWFAHRTPM | 0.0 | 0.0 |
| KTDPWFAHRTPMPKI | 0.0 | 0.0 |
| PWFAHRTPMPKIQNV | 0.1 | 0.1 |
| AHRTPMPKIQNVSSS | 0.0 | 0.0 |
| LFLLSTRQNVEGSYD | 30.5 | 17.5 |
| LSTRQNVEGSYDGAY | 0.0 | 0.0 |
| RQNVEGSYDGAYAPV | 0.0 | 0.0 |
| SYDGAYAPVLQDFRS | 1.7 | 0.1 |
| DGAYAPVLQDFRSLN | 11.1 | 2.0 |
| NNPKEWLQVDFQKTM | 12.6 | 0.0 |
| PKEWLQVDFQKTMKV | 36.8 | 44.1 |
| EWLQVDFQKTMKVTG | 57.0 | 33.3 |
| LQVDFQKTMKVTGVT | 30.7 | 13.5 |
| QKTMKVTGVTTQGVK | 0.0 | 0.0 |
| MKVTGVTTQGVKSLL | 0.0 | 0.0 |
| QKFSSLYISQFIIMY | 0.5 | 0.1 |
| FSSLYISQFIIMYSL | 0.6 | 0.0 |
| YISQFIIMYSLDGKK | 1.8 | 0.2 |
| SQFIIMYSLDGKKWQ | 4.8 | 0.5 |
| IIMYSLDGKKWQTYR | 6.0 | 1.7 |
| YSLDGKKWQTYRGNS | 0.0 | 0.0 |
| IFNPPIIARYIRLHP | 0.7 | 0.0 |
| NPPIIARYIRLHPTH | 0.4 | 0.0 |
| PIIARYIRLHPTHYS | 0.3 | 0.0 |
| IARYIRLHPTHYSIR | 1.0 | 0.0 |
| YIRLHPTHYSIRSTL | 7.9 | 2.4 |
| RSNAWRPQVNNPKEW | 0.0 | 0.0 |
| AWRPQVNNPKEWLQV | 0.0 | 0.0 |
| PQVNNPKEWLQVDFQ | 0.0 | 0.0 |
| PKEWLQVDFQKTMKV | 60.5 | 19.5 |
| EWLQVDFQKTMKVTG | 65.6 | 31.3 |
| SGYTFKHKMVYEDTL | 1.3 | 0.0 |
| YTFKHKMVYEDTLTL | 0.8 | 0.0 |
| HKMVYEDTLTLFPFS | 56.5 | 14.2 |
| KMVYEDTLTLFPFSG | 57.6 | 21.0 |
| TLTLFPFSGETVFMS | 0.0 | 0.0 |
| VVNSLDPPLLTRYLR | 2.7 | 0.4 |
| PPLLTRYLRIHPQSW | 0.0 | 0.0 |
| PLLTRYLRIHPQSWV | 0.0 | 0.0 |
| TRYLRIHPQSWVHQI | 0.0 | 0.0 |
| RIHPQSWVHQIALRM | 0.0 | 0.0 |
| TVEDGPTKSDPRCLT | 0.2 | 0.0 |
| GPTKSDPRCLTRYYS | 4.8 | 1.6 |
| TKSDPRCLTRYYSSF | 12.6 | 2.4 |
| SDPRCLTRYYSSFVN | 0.5 | 0.0 |
| PRCLTRYYSSFVNME | 0.9 | 0.1 |
| SLHAVGVSYWKASEG | 1.3 | 0.1 |
| AVGVSYWKASEGAEY | 0.0 | 0.0 |
| SYWKASEGAEYDDQT | 0.0 | 0.0 |
| ASEGAEYDDQTSQRE | 0.0 | 0.0 |
| **DRB1*04:01** | | |
| **Peptide** | **Binding Score** | **Stability Index** |
| SRPYNIYPHGITDVR | 0.2 | 0.0 |
| YNIYPHGITDVRPLY | 2.1 | 0.2 |
| YPHGITDVRPLYSRR | 0.1 | 0.0 |
| GITDVRPLYSRRLPK | 0.8 | 0.1 |
| DVRPLYSRRLPKGVK | 0.2 | 0.0 |
| PENDIEKTDPWFAHR | 0.7 | 0.1 |
| DIEKTDPWFAHRTPM | 7.6 | 0.4 |
| KTDPWFAHRTPMPKI | 8.2 | 0.5 |
| PWFAHRTPMPKIQNV | 6.4 | 0.8 |
| AHRTPMPKIQNVSSS | 1.3 | 0.1 |
| LFLLSTRQNVEGSYD | 17.9 | 2.8 |
| LSTRQNVEGSYDGAY | 15.2 | 3.2 |
| RQNVEGSYDGAYAPV | 0.0 | 0.0 |
| SYDGAYAPVLQDFRS | 0.4 | 0.0 |
| DGAYAPVLQDFRSLN | 0.7 | 0.1 |
| NNPKEWLQVDFQKTM | 1.7 | 0.1 |
| PKEWLQVDFQKTMKV | 5.1 | 0.3 |
| EWLQVDFQKTMKVTG | 26.4 | 4.8 |
| LQVDFQKTMKVTGVT | 11.6 | 0.9 |
| QKTMKVTGVTTQGVK | 6.6 | 0.5 |
| MKVTGVTTQGVKSLL | 7.8 | 0.8 |
| QKFSSLYISQFIIMY | 1.9 | 0.2 |
| FSSLYISQFIIMYSL | 1.5 | 0.4 |
| YISQFIIMYSLDGKK | 44.5 | 30.9 |
| SQFIIMYSLDGKKWQ | 38.1 | 11.8 |
| IIMYSLDGKKWQTYR | 8.3 | 0.7 |
| YSLDGKKWQTYRGNS | 0.9 | 0.1 |
| IFNPPIIARYIRLHP | 1.7 | 0.0 |
| NPPIIARYIRLHPTH | 5.0 | 3.1 |
| PIIARYIRLHPTHYS | 25.4 | 6.9 |
| IARYIRLHPTHYSIR | 61.3 | 38.8 |
| YIRLHPTHYSIRSTL | 59.3 | 61.0 |
| RSNAWRPQVNNPKEW | 18.2 | 12.4 |
| AWRPQVNNPKEWLQV | 17.7 | 10.2 |
| PQVNNPKEWLQVDFQ | 0.0 | 0.0 |
| PKEWLQVDFQKTMKV | 4.1 | 0.2 |
| EWLQVDFQKTMKVTG | 28.1 | 5.4 |
| SGYTFKHKMVYEDTL | 1.0 | 0.1 |
| YTFKHKMVYEDTLTL | 5.0 | 0.5 |
| HKMVYEDTLTLFPFS | 24.2 | 5.5 |
| KMVYEDTLTLFPFSG | 29.7 | 8.3 |
| TLTLFPFSGETVFMS | 7.3 | 0.6 |
| VVNSLDPPLLTRYLR | 1.2 | 0.1 |
| PPLLTRYLRIHPQSW | 9.6 | 0.9 |
| PLLTRYLRIHPQSWV | 12.1 | 1.4 |
| TRYLRIHPQSWVHQI | 8.2 | 0.9 |
| RIHPQSWVHQIALRM | 54.3 | 12.6 |
| TVEDGPTKSDPRCLT | 0.2 | 0.0 |
| GPTKSDPRCLTRYYS | 0.5 | 0.1 |
| TKSDPRCLTRYYSSF | 0.8 | 0.1 |
| SDPRCLTRYYSSFVN | 2.4 | 0.2 |
| PRCLTRYYSSFVNME | 9.6 | 0.7 |
| SLHAVGVSYWKASEG | 1.3 | 0.1 |
| AVGVSYWKASEGAEY | 2.0 | 0.4 |
| SYWKASEGAEYDDQT | 1.5 | 0.2 |
| ASEGAEYDDQTSQRE | 0.0 | 0.0 |
| **DRB1*11:01** | | |
| **Peptide** | **Binding Score** | **Stability Index** |
| SRPYNIYPHGITDVR | 12.0 | 0.4 |
| YNIYPHGITDVRPLY | 35.8 | 1.1 |
| YPHGITDVRPLYSRR | 46.7 | 1.2 |
| GITDVRPLYSRRLPK | 13.2 | 0.6 |
| DVRPLYSRRLPKGVK | 16.9 | 3.0 |
| PENDIEKTDPWFAHR | 2.9 | 0.1 |
| DIEKTDPWFAHRTPM | 7.3 | 0.2 |
| KTDPWFAHRTPMPKI | 0.5 | 0.1 |
| PWFAHRTPMPKIQNV | 4.9 | 0.5 |
| AHRTPMPKIQNVSSS | 0.2 | 0.0 |
| LFLLSTRQNVEGSYD | 92.0 | 23.6 |
| LSTRQNVEGSYDGAY | 0.2 | 0.0 |
| RQNVEGSYDGAYAPV | 0.1 | 0.0 |
| SYDGAYAPVLQDFRS | 0.1 | 0.0 |
| DGAYAPVLQDFRSLN | 0.2 | 0.0 |
| NNPKEWLQVDFQKTM | 3.5 | 0.1 |
| PKEWLQVDFQKTMKV | 8.6 | 0.3 |
| EWLQVDFQKTMKVTG | 157.4 | 16.4 |
| LQVDFQKTMKVTGVT | 235.0 | 62.0 |
| QKTMKVTGVTTQGVK | 0.1 | 0.0 |
| MKVTGVTTQGVKSLL | 0.1 | 0.0 |
| QKFSSLYISQFIIMY | 0.4 | 0.0 |
| FSSLYISQFIIMYSL | 0.3 | 0.0 |
| YISQFIIMYSLDGKK | 114.1 | 33.1 |
| SQFIIMYSLDGKKWQ | 100.8 | 27.9 |
| IIMYSLDGKKWQTYR | 23.3 | 1.4 |
| YSLDGKKWQTYRGNS | 23.5 | 1.1 |
| IFNPPIIARYIRLHP | 61.5 | 6.3 |
| NPPIIARYIRLHPTH | 76.5 | 6.3 |
| PIIARYIRLHPTHYS | 118.8 | 9.0 |
| IARYIRLHPTHYSIR | 150.5 | 36.2 |
| YIRLHPTHYSIRSTL | 174.3 | 30.2 |
| RSNAWRPQVNNPKEW | 0.1 | 0.0 |
| AWRPQVNNPKEWLQV | 0.1 | 0.0 |
| PQVNNPKEWLQVDFQ | 0.1 | 0.0 |
| PKEWLQVDFQKTMKV | 4.2 | 0.2 |
| EWLQVDFQKTMKVTG | 152.7 | 16.2 |
| SGYTFKHKMVYEDTL | 14.9 | 2.2 |
| YTFKHKMVYEDTLTL | 31.6 | 13.7 |
| HKMVYEDTLTLFPFS | 5.4 | 0.2 |
| KMVYEDTLTLFPFSG | 11.7 | 0.0 |
| TLTLFPFSGETVFMS | 0.1 | 0.0 |
| VVNSLDPPLLTRYLR | 0.2 | 0.0 |
| PPLLTRYLRIHPQSW | 42.2 | 5.9 |
| PLLTRYLRIHPQSWV | 62.8 | 8.8 |
| TRYLRIHPQSWVHQI | 49.4 | 5.3 |
| RIHPQSWVHQIALRM | 74.9 | 4.5 |
| TVEDGPTKSDPRCLT | 0.0 | 0.0 |
| GPTKSDPRCLTRYYS | 1.2 | 0.0 |
| TKSDPRCLTRYYSSF | 23.1 | 0.8 |
| SDPRCLTRYYSSFVN | 7.8 | 0.5 |
| PRCLTRYYSSFVNME | 20.5 | 1.2 |
| SLHAVGVSYWKASEG | 0.3 | 0.0 |
| AVGVSYWKASEGAEY | 0.3 | 0.0 |
| SYWKASEGAEYDDQT | 2.8 | 0.0 |
| ASEGAEYDDQTSQRE | 0.0 | 0.0 |
| **DRB1*07:01** | | |
| **Peptide** | **Binding Score** | **Stability Index** |
| SRPYNIYPHGITDVR | 0.0 | 0.0 |
| YNIYPHGITDVRPLY | 0.1 | 0.1 |
| YPHGITDVRPLYSRR | 0.1 | 0.1 |
| GITDVRPLYSRRLPK | 2.0 | 0.6 |
| DVRPLYSRRLPKGVK | 0.2 | 0.2 |
| PENDIEKTDPWFAHR | 0.0 | 0.0 |
| DIEKTDPWFAHRTPM | 0.0 | 0.0 |
| KTDPWFAHRTPMPKI | 4.0 | 1.2 |
| PWFAHRTPMPKIQNV | 2.7 | 3.2 |
| AHRTPMPKIQNVSSS | 0.0 | 0.0 |
| LFLLSTRQNVEGSYD | 0.3 | 0.3 |
| LSTRQNVEGSYDGAY | 0.0 | 0.0 |
| RQNVEGSYDGAYAPV | 0.0 | 0.0 |
| SYDGAYAPVLQDFRS | 0.0 | 0.0 |
| DGAYAPVLQDFRSLN | 0.1 | 0.0 |
| NNPKEWLQVDFQKTM | 0.6 | 0.1 |
| PKEWLQVDFQKTMKV | 0.0 | 0.0 |
| EWLQVDFQKTMKVTG | 0.1 | 0.1 |
| LQVDFQKTMKVTGVT | 0.1 | 0.1 |
| QKTMKVTGVTTQGVK | 0.0 | 0.0 |
| MKVTGVTTQGVKSLL | 0.0 | 0.0 |
| QKFSSLYISQFIIMY | 0.0 | 0.0 |
| FSSLYISQFIIMYSL | 0.0 | 0.0 |
| YISQFIIMYSLDGKK | 0.0 | 0.0 |
| SQFIIMYSLDGKKWQ | 0.0 | 0.0 |
| IIMYSLDGKKWQTYR | 0.0 | 0.1 |
| YSLDGKKWQTYRGNS | 0.0 | 0.0 |
| IFNPPIIARYIRLHP | 0.0 | 0.0 |
| NPPIIARYIRLHPTH | 0.2 | 0.2 |
| PIIARYIRLHPTHYS | 0.0 | 0.0 |
| IARYIRLHPTHYSIR | 0.0 | 0.0 |
| YIRLHPTHYSIRSTL | 17.2 | 20.6 |
| RSNAWRPQVNNPKEW | 0.0 | 0.0 |
| AWRPQVNNPKEWLQV | 0.0 | 0.0 |
| PQVNNPKEWLQVDFQ | 0.0 | 0.0 |
| PKEWLQVDFQKTMKV | 0.0 | 0.0 |
| EWLQVDFQKTMKVTG | 0.2 | 0.2 |
| SGYTFKHKMVYEDTL | 0.0 | 0.0 |
| YTFKHKMVYEDTLTL | 0.0 | 0.0 |
| HKMVYEDTLTLFPFS | 0.0 | 0.0 |
| KMVYEDTLTLFPFSG | 0.0 | 0.0 |
| TLTLFPFSGETVFMS | 0.0 | 0.0 |
| VVNSLDPPLLTRYLR | 0.0 | 0.0 |
| PPLLTRYLRIHPQSW | 7.0 | 8.4 |
| PLLTRYLRIHPQSWV | 1.7 | 2.0 |
| TRYLRIHPQSWVHQI | 34.0 | 40.8 |
| RIHPQSWVHQIALRM | 2.1 | 2.6 |
| TVEDGPTKSDPRCLT | 0.0 | 0.0 |
| GPTKSDPRCLTRYYS | 0.0 | 0.0 |
| TKSDPRCLTRYYSSF | 0.0 | 0.0 |
| SDPRCLTRYYSSFVN | 0.1 | 0.0 |
| PRCLTRYYSSFVNME | 0.0 | 0.0 |
| SLHAVGVSYWKASEG | 0.2 | 0.0 |
| AVGVSYWKASEGAEY | 0.0 | 0.0 |
| SYWKASEGAEYDDQT | 0.0 | 0.0 |
| ASEGAEYDDQTSQRE | 0.0 | 0.0 |
| **DRB1*15:03** | | |
| **Peptide** | **Binding Score** | **Stability Index** |
| SRPYNIYPHGITDVR | 0.2 | 0.1 |
| YNIYPHGITDVRPLY | 0.1 | 0.1 |
| YPHGITDVRPLYSRR | 0.1 | 0.1 |
| GITDVRPLYSRRLPK | 2.6 | 3.1 |
| DVRPLYSRRLPKGVK | 2.4 | 2.8 |
| PENDIEKTDPWFAHR | 0.3 | 0.0 |
| DIEKTDPWFAHRTPM | 0.1 | 0.0 |
| KTDPWFAHRTPMPKI | 0.2 | 0.0 |
| PWFAHRTPMPKIQNV | 0.2 | 0.0 |
| AHRTPMPKIQNVSSS | 0.4 | 0.1 |
| LFLLSTRQNVEGSYD | 0.2 | 0.0 |
| LSTRQNVEGSYDGAY | 0.0 | 0.0 |
| RQNVEGSYDGAYAPV | 0.0 | 0.0 |
| SYDGAYAPVLQDFRS | 0.0 | 0.0 |
| DGAYAPVLQDFRSLN | 0.1 | 0.0 |
| NNPKEWLQVDFQKTM | 0.1 | 0.1 |
| PKEWLQVDFQKTMKV | 0.0 | 0.0 |
| EWLQVDFQKTMKVTG | 24.1 | 2.3 |
| LQVDFQKTMKVTGVT | 1.5 | 0.0 |
| QKTMKVTGVTTQGVK | 0.0 | 0.0 |
| MKVTGVTTQGVKSLL | 0.0 | 0.0 |
| QKFSSLYISQFIIMY | 0.0 | 0.0 |
| FSSLYISQFIIMYSL | 0.3 | 0.1 |
| YISQFIIMYSLDGKK | 1.4 | 1.7 |
| SQFIIMYSLDGKKWQ | 14.6 | 16.4 |
| IIMYSLDGKKWQTYR | 47.8 | 57.3 |
| YSLDGKKWQTYRGNS | 0.2 | 0.0 |
| IFNPPIIARYIRLHP | 3.8 | 4.5 |
| NPPIIARYIRLHPTH | 15.2 | 18.2 |
| PIIARYIRLHPTHYS | 11.1 | 13.3 |
| IARYIRLHPTHYSIR | 103.9 | 124.6 |
| YIRLHPTHYSIRSTL | 234.7 | 281.7 |
| RSNAWRPQVNNPKEW | 0.0 | 0.0 |
| AWRPQVNNPKEWLQV | 0.2 | 0.1 |
| PQVNNPKEWLQVDFQ | 0.0 | 0.0 |
| PKEWLQVDFQKTMKV | 0.0 | 0.0 |
| EWLQVDFQKTMKVTG | 17.6 | 1.6 |
| SGYTFKHKMVYEDTL | 0.0 | 0.0 |
| YTFKHKMVYEDTLTL | 0.0 | 0.1 |
| HKMVYEDTLTLFPFS | 0.1 | 0.1 |
| KMVYEDTLTLFPFSG | 0.0 | 0.0 |
| TLTLFPFSGETVFMS | 0.4 | 0.0 |
| VVNSLDPPLLTRYLR | 2.2 | 0.1 |
| PPLLTRYLRIHPQSW | 21.7 | 26.0 |
| PLLTRYLRIHPQSWV | 10.7 | 12.8 |
| TRYLRIHPQSWVHQI | 25.4 | 30.5 |
| RIHPQSWVHQIALRM | 1.4 | 0.1 |
| TVEDGPTKSDPRCLT | 0.0 | 0.0 |
| GPTKSDPRCLTRYYS | 3.8 | 0.0 |
| TKSDPRCLTRYYSSF | 26.8 | 1.6 |
| SDPRCLTRYYSSFVN | 13.8 | 16.6 |
| PRCLTRYYSSFVNME | 72.3 | 86.8 |
| SLHAVGVSYWKASEG | 0.7 | 0.0 |
| AVGVSYWKASEGAEY | 0.1 | 0.0 |
| SYWKASEGAEYDDQT | 0.0 | 0.0 |
| ASEGAEYDDQTSQRE | 0.0 | 0.0 |
